# Investigating the Factors Affecting the Need for Unilateral Space Maintainer for First Primary Molars in Late Mixed Dentition

**DOI:** 10.1155/2022/7604144

**Published:** 2022-03-29

**Authors:** Alireza Heidari, Saeedeh Mokhtari, Mohammad Hassan Hamrah, Zahra Tavana, Mahyar Heydarigoojani, Narges Tavana

**Affiliations:** ^1^Department of Pediatric Dentistry, School of Dentistry, Tehran University of Medical Sciences, Tehran, Iran; ^2^General dental practitioner, Private, Tehran, Iran; ^3^General dental practitioner, Private, Ahwaz, Iran

## Abstract

**Background:**

Premature loss of deciduous teeth can lead to loss of space and have a negative effect on occlusion. The use of space maintainers can reduce the severity of problems such as crowding. However, the literature is controversial regarding the effects of early loss of primary first molars The aim of this study was to determine the factors affecting the need for unilateral space maintainer for the first deciduous molars in late mixed dentition. In this cross-sectional study, fifty children between 6 to 8 years who had lost a primary first molar unilaterally later than 6 months ago were randomly selected. Midline deviation, molar and canine relationships at both sides, facial growth pattern, and the amount of space loss were all assessed. Data were analyzed using SPSS version 25 via one-sample *t*-test, paired *t*-test, and linear regression (alpha = 0.05).

**Methods:**

In this cross-sectional study, 47 children aged 8 to 10 years with early unilaterally loss of first primary molar in the past 6 months were randomly selected. First, the type of occlusion based on the angle molar relationship and the growth pattern of face based on the Hall and Farkas and midline were assessed. Anterior crowding was measured. SPSS 25 program and Chi-square, *t*-test, ANOVA, and linear regression were used to analyze the data. A significance level of 0.05 was considered.

**Results:**

The results showed that the amount of space loss is 0.56 mm (maxilla = 0.54 and mandible = 0.58), which is not clinically significant, and there is no need for space maintainer. Increasing age (*p* = 0.021) and increasing the percentage ratio of facial pattern (*p* = 0.009) significantly reduced the space loss and increased the duration of tooth loss (*p* = 0.002), and molar relationship in the control side (*p* = 0.05) and increasing the canine to lateral distance (*p* = 0.016) significantly increased the space loss. Other factors such as crowding, midline deviation, and canine relationship on the control side did not have significant effects on space loss.

**Conclusion:**

Space loss due to extraction of the first primary molars in late mixed dentition was neither statistically nor clinically significant. However, in cases of severe crowding, the vertical growth pattern of the face, and molar relationship, further studies are needed, and follow-up of patients is recommended.

## 1. Introduction

Various environmental and morphogenic factors manage the occlusal development, and any disturbance or aberration in any of these factors might adversely affect the occlusion. Of all these factors, the presence of primary teeth is of utmost importance because when they are exfoliated physiologically, the alveolar growth takes place, providing adequate space for successful accommodation of permanent teeth [1].

Premature extraction of deciduous teeth might result in space loss in the developing dentition, leading to malocclusion in permanent teeth. The etiology of malocclusion is complicated, consisting of genetic, hereditary, and environmental factors [2, 3]. There is a consensus that premature extraction of deciduous teeth might result in unfavorable drifts of deciduous or permanent teeth or both dentitions. This finally decreases the arch length required for permanent teeth, exacerbating the patient's malocclusion, crowding, rotation, ectopic eruption, extraction of the antagonist tooth, crossbite, increased overjet and overbite, inappropriate molar relationship, craniofacial growth disturbances, and the impaction of permanent teeth in particular [3, 4].

Deciduous molar teeth are frequently lost due to caries or infection unilaterally or bilaterally. Although the premature loss of the deciduous second molar tooth significantly increases the chance of space loss, requiring using a space maintainer [5], previous studies have reported contradictory results about the effects of the premature loss of deciduous first molar teeth. Most studies have shown that the loss of space during the first 4–6 months after extraction is associated with the drift of deciduous canine teeth and permanent incisors towards the edentulous spaces in both arches [6, 7]. Some studies have reported slight mesial drift of maxillary deciduous second molar teeth [8]. Some other studies have shown that the space loss might lead to the impaction of permanent canines, especially in the upper jaw [9]. However, some studies have reported that the premature loss of deciduous first molars does not significantly decrease the arch's width, length, and circumference [10, 11]. A systematic review showed that the loss of deciduous first molar teeth results in 1.5 mm space loss on each side in the mandible and 1.0 mm on each side of the maxilla, which is not important clinically [12].

Generally, reports indicate that patients in the full primary and mixed dentition periods undergo less space deficiency with proper contacts between the cusps of permanent teeth [13]. Therefore, some researchers question the need for space maintainers when deciduous first molar teeth are lost prematurely under these conditions [8, 9, 13]. However, in general, maintaining space in children is considered important and recommended in the mixed dentition period [14, 15].

Therefore, considering the importance of space maintenance in managing developing occlusion and the lack of a standard protocol for determining the need for a space maintainer, the present study aimed to determine the need for a space maintainer after unilateral extraction of deciduous first molar teeth in children in the late mixed dentition period.

## 2. Materials and Methods

The present cross-sectional study's protocol was approved by the Ethics Committee of Tehran University of Medical Sciences under the code IR.TUMS.DENTISTRY.REC.1398.113.653; children were examined in a well-lit room using a mirror and swap by a trained dentist; and among them, 47 children who had the inclusion criteria listed below were chosen.

### 2.1. Inclusion Criteria

The inclusion criteria are as follows:

- Completing an informed written consent form by the patient's parents or legal guardians
- The child's compliance and agreement to carry out evaluations
- The presence of fully erupted maxillary and mandibular permanent first molar and lateral and central incisors
- The presence of all the other teeth at least in the previous six months
- The absence of interdental caries or restored interdental caries
- No use of any appliance

### 2.2. Exclusion Criteria

The exclusion criteria are as follows:

- No consent to be included in the study
- Lack of the ability or consent to cooperate
- Children not yet in the late mixed dentition period (unerupted maxillary and mandibular permanent incisors and permanent first molars not in the occlusion)
- Children having lost a tooth in addition to the deciduous first molar tooth in the same jaw

**Figure 1:**
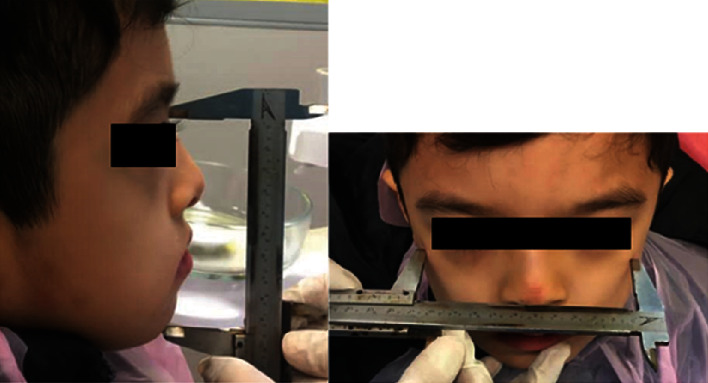
Method of measuring maximum face width and maximum face height.

After referring the children and obtaining informed consent from the parents, the following items, in addition to entering the age of the child, were measured and recorded by a pediatric dentistry resident:

1. The patient's age was calculated according to the year, taking into account the date of birth
2. The time elapsed since the tooth was extracted in terms of months was obtained through the patient's parents
3. Molar relationship of the parasite (class I, II, and III) by assessing the position of the upper permanent molar's mesiobuccal cusp relative to the lower permanent molar's buccal groove. So there were three groups of molar relationships: class I, class II, and class III [16]
4. The relationship of the canine (class I, II, and III) was determined by evaluating the position of the cusp tip of the deciduous canine relative to the mandibular canine, so that if the cusp tip of the canine is in the embrasure between the deciduous canine and the first deciduous molars, the first premolar had a class I relationship, and if it was more mesial or more distal, it had a canine relationship, Cl II, and Cl III, respectively [17]
5. The Hall and Franks method was used to determine each child's facial growth pattern [18]. With the child at rest, from the frontal view, the ratio of the bizygomatic width of the zygomatic bone (BZM) measured with a caliper (with a range of 0 to 150 mm with an accuracy of 0.02 mm (Mitutoyo, Japan)) to the maximum facial length (MFL) was used to determine the facial growth pattern. Then, the number obtained in one of these three categories of facial growth pattern was leptoprosopic with a ratio of 75% or less, mesoprosopic with a ratio of 76% to 79%, and europrosopic with a ratio of 80% or more (Figure 1)
6. Determination of the presence or absence of midline shift, clinically and measuring the amount of deviation if present, was done clinically. To investigate the deviation of the dental midline from the facial midline, the nasion and pogonion points were connected using a dental floss, and this line was considered as the facial midline. The midline between the two central incisors in the jaw was compared with the facial midline, and the midline alignment or deviation was recorded

After molding the relevant jaw and preparing the cast, the following items were examined:

(7) Determining the distal movement of the canine tooth independent of other anterior teeth towards the space obtained from the extraction of the first deciduous molars by comparing the distance between the mesial surface of lateral teeth to the distal surface of canine (from contact point) on both sides
(8) Determination of anterior crowding was performed by Little criterion [19] and by measuring the total overflow resulting from crowding in the distance from canine to canine. The resulting number was placed in each of these groups: no crowding, 0 mm; light crowding, 1 to 3 mm; medium crowding, 4 to 6 mm; severe crowding, 7 to 9 mm; and very severe crowding, 10 mm

The data were analyzed with SPSS 25, using the chi-squared test, *t*-test, ANOVA, and linear regression analysis. Statistical significance was set at *P* < 0.05.

## 3. Results

Forty-seven 8–10-year-old children with a mean age of 9.08 ± 0.58 years were included in the present study. The majority of the participants (66%) were 9 years old. Approximately 21.3% and 12.8% of the children were 10 and 8 years old.

Twenty-five cases examined belonged to the maxilla, and 22 belonged to the mandible.  Table 1 presents the descriptive data of the variables used in the present study.

For dependent variables, such as midline deviation and canine movement, we had this result.

Regarding the midline deviation, in 95.7% of the samples, no midline deviation was observed, while in 4.3% of them, the midline deviated towards the extraction area. But according to one-sample *t*-test analysis, this number was not significant (*P* value: 0.160).

In 78.7% of the samples, the canine tooth did not move independently of the other incisors, while in 21.3% of the samples, the canine deciduous tooth, independent of the other anterior teeth, moved into the extraction space within range 2.0. mm to 0.7 mm, and its value was significant with *P* value 0.002 (one-sample *t*-test).

Out of 21.3% of the sample with independent movement of the canine tooth towards the extraction side, by comparing the amount of space loss and the amount of independent movement of the canine in the extraction side with and without midline deviation, in 5 cases, space loss occurred more from anterior. In other cases, due to the lack of midline deviation and the lack of independent movement of the canine, the space seems to have been lost from posterior side. Should we want to mention separately from the 22 mandibular cases, in 5 cases, more space was lost from the anterior and in the remaining 17 cases from the posterior.

In ANOVA one-way analysis, independent distal movement of deciduous canine teeth in the samples had no significant relationship with facial growth pattern (*P* value =0.126) and crowding (*P* value =0.923) but had a significant relationship with midline deviation (*P* value =0.05). However, the number of cases with midline deviation was only 3.

Over time, from 6 to 24 months, the amount of space loss increases. The relationship between space loss and duration of tooth loss is significant, (*r*) = 0.4, *P* = 0.006 (Pearson's correlation). The maximum amount of space loss occurred within 8 to 18 months after extraction and was in the range of 0.3 to 0.9 mm.

In the following, we explain the effect of independent variables of arch, facial growth pattern, crowding, molar relationship, canine relationship, and age on the average space loss, respectively. Finally, to evaluate the effect of independent variables on space loss, multivariate analysis was applied.

The difference in space loss between maxilla and mandible with *P* value: 0.7 was not significant, in which the maxilla was 0.53 mm on average, and in which the mandible was 0.58 mm, and space was lost.

The data analysis showed that over time, from 6 months to 24 months, the lost space increased, and the relationship between the space loss and the time since losing the teeth was direct and significant (*P* = 0.006, Pearson's correlation coefficient (*r* = 0.4)).

In evaluating the effect of facial growth pattern on the amount of space loss, the mean space loss in the leptoprosopic growth pattern was 0.81 ± 0.65 mm and greater than 0.76 ± 0.45 and 0.36 ± 0.44 mm in the mesoprosopic and euryprosopic patterns, respectively. The difference between the leptoprosopic and euryprosopic patterns was significant (*P* = 0.048). However, the differences between the leptoprosopic and mesoprosopic facial growth patterns (*P* = 0.98) and between the mesoprosopic and euryprosopic patterns (*P* = 0.075) were not significant.

Evaluation of crowding showed that 59.6% of the samples did not have crowding, 29.79% had mild crowding, 8.51% had moderate crowding, and only one person (2.1%) had severe crowding. The means of crowding in the maxilla and mandible were 1.21 and 0.71 mm, respectively, with no significant difference (*P* = 0.26). The most severe crowding was detected in the maxilla, with a space loss of 1.5 mm due to tooth extraction. However, of all the spaces lost, the most significant space loss was detected in the arches without crowding with 1.9 mm (Table 2).

The effect of molar relationship on the amount of space loss on the extraction side has been shown in Table 3.

The average amount of space loss in the maxilla in the canine relationship of class I was 0.486 mm, class II was 0.4 mm, and class III was 0.85 mm. In the mandible, these values were obtained in the canine relationship of class I, 0.602 mm, and class II, 0.39 mm. In multivariate regression analysis, the canine relationship had no significant relationship with the amount of space loss (*P* value =0.279).

Examining the age of the samples, it was seen that with increasing age, the amount of space loss decreases, so that at 8 years old 0.68 mm, at 9 years old, 0.58 mm and at 10 years old 0.39 mm of space On average was lost, all of which show a significant relationship.

According to Table 4, aging (*P* = 0.021, *B* = −0.265) and increased percentage of facial pattern (*P* = 0.009 and *B* = −2.45) significantly decreased space loss. Increased time since tooth loss (*P* = 0.002, *B* = 0.04), molar relationship on the intact side from Cl I to Cl III (*P* = 0.05, *B* = 0.185), and an increase in the distance from the canine to the lateral incisor teeth (*P* = 0.016, *B* = 0.71) significantly increased space loss. Other factors, including jaw type, crowding, midline deviation, and canine relationship on the intact side, did not significantly affect tooth loss.

## 4. Discussion

The aim of this study was to determine the factors affecting the need for unilateral space maintainer for the first deciduous molars in late mixed dentition. The overall mean space loss was 0.56 mm (0.54 mm in the maxilla and 0.58 mm in the mandible).

A systematic review showed that the loss of primary first molars resulted in a space loss of 1.5 mm on each side of the mandible and 1.0 mm on each side of the maxilla. It was concluded that such space loss was not important clinically [21]. However, some studies have recommended space maintainers to decrease the incidence and severity of malocclusion after premature tooth loss [4, 20, 21].

Although there is not much controversy over the need for a space maintainer when the deciduous second molar is lost, there is confusion about the need for the clinical management of space when the deciduous first molar is lost [22, 23].

Multivariate analysis of the significant factors in the present study showed that the time elapsed since the extraction, distal movement of the canine on the tooth extraction side, the facial growth pattern, and the child's age significantly affected the space loss.

The time elapsed since the extraction of the tooth. Although most studies have shown that the highest space loss occurs in the first 6–12-month period after extraction, the present study showed that additional space is lost over time after tooth extraction, which might be explained by approaching the early mixed dentition period [6, 7]. At this stage, some permanent teeth are erupting, increasing the odds of using the existing space. This is evident for the claim that in the late mixed dentition, especially at older ages, the time elapsed since the extraction is not an essential factor for space loss, on the condition that the tooth has really been extracted in the late mixed dentition period.

The most space loss occurred during 8 to 18 months after the extraction. The reason for this is probably the tendency of the teeth to move mesially under the influence of occlusal forces, which has a cumulative effect on the space loss over time. So that in cases where 18 months have passed since the extraction, the amount of space loss reaches an average of 0.9 mm. However, in the late mixed dentition, especially in the older ages of this group, the time elapsed since tooth extraction is not an important factor in space loss, as in the present study, it was also shown that at older ages, the average rate of space loss was significantly lower. It has been suggested that with increasing age, bone density increases and the movement of adjacent teeth becomes more difficult. Kisling et al. [24] concluded that there was no need to place a space maintainer after the emergence of the first permanent molar [25]. Richardson also emphasizes that the effect of variables on the space loss before the emergence of the first permanent molar is greater than after its emergence. However, Alexandera [18] stated that the variables of age and stage of tooth growth, when the face is growing, cannot determine the need for a space maintainer. In the vertical growth pattern in the present study, the leptoprosopic growth pattern exhibited the most significant space loss (>1 mm), with a mean of 0.8 mm. However, the space loss in the mesoprosopic (mean = 0.76 mm) and euryprosopic (mean = 0.36) growth patterns was less than 1 millimeter, with the least space loss in the mesoprosopic growth pattern. Multivariate analysis showed that the facial growth pattern significantly affected space loss too; a decrease in growth pattern ratio increased space loss percentage, indicating that space loss was significantly higher in the leptoprosopic growth pattern. Alexandera et al. [18] showed that the facial growth pattern in children >7 years of age, similar to molar occlusion patterns, can affect the need for a space maintainer when the primary first molar is lost prematurely. They showed that persons with leptoprosopic facial form and end-on molar occlusion were significantly different in space deficiency than the control group. Children with mesoprosopic and euryprosopic growth patterns and Cl I molar occlusion did not exhibit any significant difference in space loss. Therefore, they reported that it appears that patients with a hypodivergent growth pattern and Angle Cl I occlusion do not seem to need a space maintainer. In these patients with an end-on molar relationship in the maxilla, no space maintainer is required in the upper jaw, while a space maintainer is strongly recommended in the lower jaw due to the early loss of the deciduous first molar tooth. In patients with a hyperdivergent facial growth pattern and Cl I molar relationship, too, a space maintainer is recommended in the upper jaw and lower jaw (more emphatically). In patients with a vertical facial growth pattern and end-on molar relationship, too, a space maintainer has been recommended in the upper jaw (more emphatically), with a space maintainer being considered in the lower jaw.

Alexandra [18] observed that the facial growth pattern in children over 7 years of age, similar to molar occlusion, can affect the need for space maintainer in cases of early loss of the first primary molars. He showed that people with a leptoprosopic facial growth pattern and an end-on molar relationship had significantly more space loss than other groups. Children with mesoprosopic/euryprosopic facial form and class I molar occlusion did not show any significant difference in space loss, although space loss in patients with mesoprosopic/euryprosopic facial form with end-on molar relationship is only in mandible. It was significant that it has been seen that in the vertical growth pattern, orthodontic movement of teeth is easier, which can be the result of weak muscles with reduced size tonicity [26].

So it may be assumed that space loss can occur more in persons with vertical growth pattern because adjacent teeth drifting occurs more easily. In our study, the highest mean of space loss occurred in the upper jaw of a patient with class III molar relationship, leptoprosopic growth pattern and severe crowding, and in the lower jaw in a patient with class II molar relationship and leptoprosopic growth pattern without crowding. Of course, it should be noted that only 2 of the samples had a class III molar relationship on the control side, and the effect of the molar relationship on the space loss needs further investigation.

### 4.1. Distal Movement of the Canine

Most studies have accepted that with the early loss of the first mandibular primary molars, in addition to the mesial movement of the posterior teeth into the extraction space, distal movement of the deciduous canines also occurs [27, 28], but in the maxilla, the mesial movement of the second primary molars into the extraction space will be the predominant mechanism [27]. After tooth extraction, the main cause of adjacent tooth drift is the regeneration of transseptal fibers, which can lead to loss of space both anteriorly and posteriorly. Other possible causes include forces from muscles, especially the lips and cheeks, which can lead to distal drift of the canine tooth [29]. The mandibular canine appears to move more distally than other anterior teeth, leading to midline deviation because there is no obstruction on the distal side. Mandibular occlusion support may reduce the distal movement of the anterior teeth and maxillary canine. Another reason may be the force exerted by the mentalis muscle in excess of the force on the mandibular canine, whereas the maxilla lacks such force.

To show the distal movement of the canine tooth, due to the lack of information before the extraction time, we had to compare the distance between the midpoint of the mesial surface in the permanent lateral to the midpoint of the distal surface of the primary canine tooth with the same distance on the control side.

Based on the information obtained from the distal movement of the canine and comparing it with the amount of space loss, we found that in 23% of the cases that lost the first mandibular primary molar, the extracted tooth space is lost more due to the distal movement of the canine than the mesial movement of the posterior teeth.

Multivariate test showed that distal movement of the canine significantly caused space loss on the side of the extracted primary molars, which was mainly in the mandible. Independent distal movement of deciduous canine in the samples had no significant relationship with facial growth pattern and crowding. The number of cases of midline deviation in the present study was only 3, but there was a significant relationship (*P* value =0.05) with the independent movement of canine teeth.

Nowak believes that space is often lost from the anterior side with the loss of the mandibular first primary molar tooth [30]. The study by Lin et al. also confirms the effect of canine distal movement in cases of early extraction of the first primary molars [31]. In our samples, due to the patient's age (8 to 10 years), the secondary spacing that occurs due to the pressure of lateral growth to the canine in the mandible [32] is completed with the complete growth of permanent laterals. On the other hand, the intercanine width stabilizes at the age of 8 [33], thus reducing the possibility of distal movement of the canine tooth and the space loss in the anterior region. Although the effect of canine movement on the space loss was significant, the numerical value (0.2 to 0.7 mm) of space loss is not sufficient to justify the prescription of space maintainer and therefore is clinically significant.

In the present study, in multivariate analysis, other factors such as crowding, midline deviation, and jaw type did not have a significant effect on space loss.

#### 4.1.1. Crowding

The effect of anterior crowding on the loss of the first primary molars has not been specifically studied in other studies. In the present study, the highest mean space loss was reported for severe crowding and the lowest for arches without crowding. However, because most of our samples (59.6%) were in the group without crowding, and only one case had severe crowding, no significant correlation was observed, and the results are not generalizable. Some studies in this field, such as Richardson's research, show more space loss in crowded arches than in spaced arches [25].

There was no significant relationship between the mean crowding in the upper and lower jaws and the space loss in the two jaws in the present study. Although there was severe anterior crowding in the upper jaw with 1.6 mm of space loss after extraction, the highest space loss occurred in the jaw arch without crowding with 1.9 mm, which can be explained in different ways. First, most samples were in the group without crowding, making the comparison of these four groups less reliable. Second, other variables might have a role in space loss, including the time elapsed since the extraction, which brings the samples closer to the previous dental period. Third, the patient might have crowding and have compensated it with leeway space, resulting in midline deviation.

Rock et al. [33] concluded that in dental arches with crowding, the extraction of the contralateral deciduous first molar tooth might be necessary to improve a deviated middle (off). They showed that after extracting the deciduous first molar tooth, three situations might ensue: (1) Without midline deviation, in which tooth extraction on the contralateral side is not necessary; (2) midline deviation along with complete space loss, necessitating orthodontic treatmen; and (3) midline deviation along with some space remaining in the extraction site.

However, in the present study, since the midline had not deviated in the majority of cases (with crowding, even severe crowding, and without crowding), it does not appear that crowding can justify the prescription of a space maintainer. As mentioned previously, in the present study, the mean difference in space loss between the upper and lower jaws was negligible at 0.004 mm, and the two jaws did not exhibit a significant difference in space loss. However, several studies have shown that space loss in the mandible occurs earlier than the maxilla [34, 35], which might be attributed to the large leeway space in the mandible than the maxilla, resulting in the late mesial shift of permanent first molars in the use of quadrant spaces [36]. However, it appears that the similarity of the lost space in the two jaws and the smallness of this space, in general, prevent space loss; therefore, there is no need for a space maintainer due to the complete occluding of permanent molar teeth and the presence of overjet when the permanent anterior teeth have erupted.

The results of the present study were consistent with the results of many studies considering the variables of facial growth pattern, molar relationship, and age of the patient, but by considering the variable of crowding (which was not specifically evaluated in other studies), new results were obtained. However, the results of this study cannot be generalized for some reasons, such as limited number of samples, examination after 6 months of tooth extraction, and lack of equal distribution of samples in different groups of variables such as facial growth pattern, molar-canine relationship, and crowding, and further investigation is needed.

## 5. Conclusion

According to the present study, the conclusions are as follows:

1. Factors affecting the amount of space loss and thus judging on the need or not the need for the space maintainer in the late mixed dentition period were as follows: facial growth pattern, patient age, and the time elapsed since the extraction of the tooth and molar relationships
2. Crowding factors and the type of canine relationship and the type of arc involved did not have a significant relationship with the amount of space lost
3. Loss of space in both jaws often occurred due to the mesial movement of the posterior teeth, although in the mandible, the distal movement of the primary canine also significantly caused the loss of space
4. Extraction of the first deciduous molars had no significant relationship with midline deviation

## Figures and Tables

**Table 1:** The description of the variables used in the present study.

Independent variables	Min	Max	Mean	SD
The time elapsed since tooth extraction_(month)_	6	24	11.85	5.805
Facial width-to height ratio	0.72	0.97	0.8073	0.06660
Crowding_(mm)_	0.00	6.30	0.9809	1.57130
Space loss_(mm)_	0	1.9	0.5591	0.52889
Midline deviation_(mm)_	0	0.8	0.032	0.1534
Canine movement towards the extraction space_(mm)_	0.00	0.90	0.1070	0.22771

**Table 2:** The amount of space loss in each jaw based on the amount of crowding.

Arch	Crowding amount	Frequency	Average space loss_(mm)_	Standard deviation
Maxilla	Without crowding	15	0.39	0.47
	Mild	5	0.70	0.30
	Moderate	4	0.62	0.45
	Severe	1	1.50	

Mandible∗	Without crowding	13	0.66	0.66
	Mild crowding	9	0.47	0.55

∗In our study, there were no cases of extraction of first primary molar in mandibular arch with moderate and severe crowding.

**Table 3:** The effect of molar relationship on the control side on the average amount of space lost on the extracted tooth side.

Arch with extraction	Molar relation ship	frequency	Average space loss	Standard deviation
Maxilla	Cl I	18	0.45	0.46
	Cl II	3	0.33	0.32
	Cl III	4	1.07	0.30

Mandible	Cl I	17	0.50	0.58
	Cl II	5	0.86	0.59
	Cl III	0	0	0

**Table 4:** Multivariate analysis using the backward linear regression to show the simultaneous effect of independent variables on the space loss.

Variables	*β* regression coefficient	Beta	*P* value
Age_(years old)_	-0.265	-0.293	0.021
Tooth extraction time_(month)_	0.038	0.421	0.002
∗Molar relationship on the control side	0.185	0.222	0.051
Facial pattern	-2.455	-0.309	0.009
Canine movement _(mm)_	0.713	0.307	0.016
Crowding			0.760
Canine relationship on the control side			0.279
Midline			0.576
Jaw			0.723

## Data Availability

The data are available from the corresponding author on reasonable request.
